# Postcode Lotteries in Public Health - The NHS Health Checks Programme in North West London

**DOI:** 10.1186/1471-2458-11-738

**Published:** 2011-09-28

**Authors:** Clare EM Graley, Katherine F May, David C McCoy

**Affiliations:** 1 Public Health Directorate, NHS Inner North West London, 15 Marylebone Road, Westminster, NW1 5JD, UK

## Abstract

**Background:**

Postcode lotteries in health refer to differences in health care between different geographic areas. These have been previously associated with clinical services. However there has been little documentation of postcode lotteries relating to preventative health care services. This paper describes a postcode lottery effect in relation to the NHS Health Checks Programme (a national cardiovascular screening programme in England) in eight PCTs in the North West sector of London.

**Methods:**

A descriptive cross-sectional analysis of the Health Checks Programme was carried out in eight PCTs in North West London using a structured data-collecting instrument.

**Results:**

The analysis found variation in the implementation of the national Health Checks Programme in terms of: the screening approach taken; the allocated budget (which varied from £69,000 to £1.4 million per 100,000 eligible population); payment rates made to providers of Health Checks; tools used to identify and measure risk of cardiovascular disease and diabetes; monitoring and evaluation; and preventative services available following the health check.

**Conclusions:**

This study identifies a postcode lottery effect related to a national public health programme. Although it is important to allow enough flexibility in the design of the Health Checks Programme so that it fits in with local factors, aspects of the programme may benefit from greater standardisation or stronger national guidance.

## Background

Postcode lotteries in health refer to variations in health care between different geographical areas that appear arbitrary and un-linked to health need. The term is usually associated with clinical services and has been used in relation to out-of-hospital cardiac arrest response rates [1], access to cancer treatments [2], access to surgery [3], and access to specialist palliative care [4]. However, there has been little documentation of postcode lotteries related to public health programmes.

This paper describes a 'postcode lottery' effect in relation to the NHS Health Checks Programme which was introduced in 2009 by the Department of Health (DH) to provide population-wide screening for cardiovascular disease (CVD). Vascular diseases make up a third of the difference in life expectancy between spearhead areas and the rest of England [5] the Health Checks Programme therefore offers an opportunity to reduce health inequalities. Adults aged between 40 and 74 (with no prior diagnosed vascular disease) are screened to assess their risk of a cardiovascular event in the next 10 years and are provided with a combination of advice and medication as required. The national programme aims to achieve full coverage of the target population over a five year period.

The funding and delivery of the programme has been delegated to Primary Care Trusts (PCTs) in England, public sector organisations that are responsible for commissioning health services for the population of a defined catchment area. Although national guidelines exist to help ensure some degree of quality assurance, consistency and standardisation (Table 1) [6], PCTs are expected to design and deliver the programme in a way that suits local circumstances. The freedom that PCTs have in the funding, design and implementation of their local Health Checks Programme allows for considerable variation to emerge across the country. In order to examine the nature and scale of this variation, we carried out a descriptive analysis of the Health Checks Programme in each of the eight PCTs in North West London.

**Table 1:** National Health Checks Guidelines

**1**.	People aged 40 to 74 who have not been diagnosed with heart disease, stroke, kidney disease or diabetes will be invited to have a check once every 5 years.
**2**.	It is expected that one fifth of the eligible population will be invited for a check each year.

**3**.	Health checks must be carried out face to face in an area that allows for privacy.

**4**.	The person carrying out the check must have had adequate training.

**5**.	The health check should be carried out with suitably calibrated equipment.

**6**.	The health check should consist of: clinical history (age, gender, smoking status, family history of Coronary heart disease and ethnicity); measure of Body Mass Index (BMI), blood cholesterol and blood pressure (BP); and a screening test for diabetes.

**7**.	CVD risk should be calculated using an appropriate risk engine calculator as advised by NICE (either Framingham or QRISK2).

**8**.	Everybody who undergoes a health check should have their risk conveyed to them.

SOURCE: (DH, 2008)

## Methods

A cross-sectional study of the Health Checks Programme in each PCT in North West London was conducted between 2^nd ^September and 16^th ^September 2010. Data were collected using a structured questionnaire which covered the following aspects of the Health Checks Programme: eligibility criteria; approach used to ensure population coverage; funding and associated payment structures; equipment and screening instruments; CVD risk calculation tool used; additional interventions; monitoring and evaluation; and advice, treatment and referrals (this questionnaire used can be found in additional file 1). The questionnaire was completed by the Health Check lead of each PCT. Any additional data or clarifications were obtained via email. Consent to publish the data received was obtained from Health Check Leads and Directors of Public Health. The data collected for this study are available on request.

## Results

### Eligibility

Most PCTs followed the national guidance on eligibility although in the first year of their programme, two PCTs had extended the lower age limit to 35 years for individuals from high risk communities, while one PCT had reduced the upper age limit to 70 years. However, inconsistency in the way 'pre-existing CVD' was defined resulted in differences in eligibility: national guidance had not specified whether patients with hypertension and atrial fibrillation, or patients already on statin medication should be included or excluded from the Health Checks Programme. Since this study was conducted, further clarification has been provided on the definition of 'pre existing CVD' which now includes those who have been diagnosed with coronary heart disease, chronic kidney disease, diabetes, hypertension, atrial fibrillation, transient ischemic attack, familial hypercholesterolemia, heart failure, peripheral arterial disease or peripheral vascular disease or those prescribed statins.

All eight PCTs defined their base population according to individuals registered with their local GP practices; however, two PCTs also allowed patients who were resident in the borough but registered with GP practices elsewhere.

### Approach to Achieving Population Coverage

A systematic approach to achieving population coverage, primarily through the provision of health checks in GP surgeries, was adopted by all the PCTs. However, in three PCTs, the eligible population was stratified into five groups (one for each of the 5 years allocated to achieve full coverage) according to an estimated risk of CVD based on markers of CVD obtained from GP records. The group with the highest risk of individuals were prioritised and invited to have a health check in the first year of the programme, followed each year by the group with the next highest set of risks. In the other five PCTs, patients were allocated into groups randomly.

In five PCTs, health checks were also carried out opportunistically in a number of settings. These settings included community pharmacies (in four PCTs), and community events at locations such as faith centres, workplaces and shopping centres (in five PCTs). Overall, three PCTs offered health checks *only *in GP practices. One of these previously carried out health checks opportunistically at community events in the first year of the programme this was stopped in the second year. Four PCTs also offered health checks in GP practices, pharmacies *and *at community events (see Table 2). Health checks carried out in community pharmacies and at outreach events tended to be used by PCTs as a means of getting to hard-to-reach or deprived individuals.

**Table 2:** Eligibility Criteria and Approach Taken by PCTs

PCT	Health Check Approach	Setting	Eligibility Criteria
**1**	Systematic	GP	40-74
**2**	Systematic Opportunistic	GP	Year 1: 35 - 74 who are at 20% or greater risk of developing a CVD event within 10 years and who do not already have established disease. Year 2: 40 - 74 with BMI of greater than or equal to 30 without established disease.
**3**	Systematic Opportunistic	GP Community events Pharmacies	Year 1: 35-74 year olds who had an estimated risk of developing CVD greater than 20% who do not have existing disease. Year 2: 40 -74 year olds who had an estimated risk of developing CVD greater than 20% and opportunistic for hard to reach groups.
**4**	Systematic	GP	40-74 year-olds without pre-existing diagnosis of any of the following conditions: hypertension, atrial fibrillation, previous TIA or stroke, hypercholesterolemia, heart failure, PAD, CHD, diabetes, chronic kidney disease (stage3-5).
**5**	Systematic Opportunistic	GP Pharmacies	People who are aged 40 - 74 who have not been diagnosed with or treated for a vascular disease and who are either resident in or registered with a GP.
**6**	Systematic Opportunistic	GP Community events	40 and 74 and not on a related disease register.
**7**	Systematic Opportunistic	GP Community events Pharmacies	40 -74 year olds without pre existing diagnosis of vascular disease. Age range extended to 35 for homeless people and people with mental health needs.
**8**	Systematic Opportunistic	GP Community events Pharmacies	40 -74 year olds without pre existing diagnosis of vascular disease. Must be registered with a GP in the borough.

### Funding and Payment Structures

There was considerable variation in the budget made available by individual PCTs for the Health Checks Programme, ranging from £69,000 to £1.4 million per 100,000 eligible population (see Table 3). This allocated budget mainly covered the cost of buying the required equipment, paying providers to carry out the Health Check, marketing the service and managing the programme. Only one PCT (number 4) reported that their budget included a behaviour change intervention.

**Table 3:** Budget and Fee Paid per Health Check by PCT

PCT	2010-11 allocated budget for health checks[b]	2010-11 budget standardised per 100,000 eligible population	Estimated eligible population (40- 74 year olds)	Rank of deprivation [c]	CHD Mortality rate in people under 75[d]	Fee per General Practice HC	Fee per Pharmacy HC
**1**	£333,000	£322,349	103,304	24	45.0	£25	-
**2**	£350,000	£298,000	117,226	61	53.8	£16*	-
**3**	£289,000	£532,200	54,290	31	40.1	£16	£30
**4**	£58,000	£69,172	83,848	184	33.6	£13	-
**5**	£305,000	£315,744	96,597	130	44.8	£40	£30
**6**	£150,000	£177,127	84,685	92	45.4	£20	-
**7**	£911,162	£1,404,595	64,871	98	23.2	£37	£45
**8**	£535,000	£681,320	78,524	75	28.9	£35	£35

*£2 extra if phlebotomy included

The amount paid to individual providers also varied, with the price paid to GP practices ranging from £13 to £40 per health check. The cost of a health check completed in a pharmacy also varied between the highest and lowest paid by £10. It is not however appropriate to compare the amount paid to GPs with the amount paid to pharmacies because of the different ways that they are funded and contracted more generally by the NHS.

### Equipment and Screening Instruments

In most PCTs, health checks conducted in GP surgeries sent blood samples for glucose and cholesterol testing to a laboratory. Only two PCTs used Point of Care Testing (POCT) in the GP setting.

Of the six PCTs conducting Health Checks in non-GP settings, POCT equipment was used to analyse cholesterol and glucose levels using one of two recommended kits: cardiocheck analysers (BHR) and Cholestech LDX analysers (Alere). There are a number of differences between these two options, including differences in sensitivity and specificity (see Table 4) and cost (with the cardiocheck analyser costing approximately 50% and 30% cheaper than the LDX in terms of start-up and running costs respectively). However, there was an even split in the number of PCTs using each option.

**Table 4:** Sensitivity and Specificity of Point of Care Testing Equipment

	Sensitivity		Specificity	
Result	Cardiocheck[e]	LDX[f]	Cardiocheck	LDX
**TC/HDL Ratio>5.0**	90%	80%	74%	99%
**Total Cholesterol >5.2 and or TC/HDL ratio>5.0**	98%	94%	55%	89%
**Total cholesterol >6.5 and TC/HDL ratio >5.0**	93%	80%	85%	98%

There is no national consensus on how best to identify people with impaired glucose tolerance or undiagnosed diabetes. However, national guidance is that individuals with a high BMI (>30 (or >27 if Indian, Pakistani, Bangladeshi, other Asian or Chinese)) or hypertension (>140/90) should undergo an Hb1AC or fasting blood glucose (FBG) test. However, only one PCT followed this guidance. In five PCTs a FBG was encouraged in *all *individuals who received a health check in a GP setting, with random blood glucose (RBG) test was allowed as an alternative in two of these PCTs. Two PCTs used a non-invasive risk assessment tool (FindRisc and QDScore) to determine who should receive further testing for diabetes (See Table 5). In community settings where health checks were offered on an opportunistic basis using POCT all four PCTs used random, non-fasting capillary blood samples to test for diabetes in all patients receiving a health check, and one used FindRisc.

**Table 5:** Screening Tools Used During the Health Check

PCT	Diabetes screening tool (GP)	Diabetes Screening Tool (Community)	CVD risk calculator used	Additional Screening tests
**1**	BMI>30 or high BP		QRISK 2	
**2**	Fasting blood glucose		QRISK 2 or JBS 2	AF
**3**	Random/ Fasting Capillary blood glucose	Random capillary blood glucose	JBS 2	Alcohol Physical Inactivity
**4**	QDRISK		QRISK 2	Alcohol
**5**	Fasting venous blood glucose	Random capillary blood glucose	QRISK 2 or JBS 2	Alcohol Physical Inactivity
**6**	Fasting venous blood glucose	Random capillary blood glucose	QRISK 2 or JBS 2	AF Alcohol Physical Inactivity
**7**	FindRisc	FindRisc	QRISK 2	Alcohol Physical Inactivity
**8**	Random/Fasting venous blood sample	Random capillary blood glucose	QRISK 2	AF Alcohol

### CVD Risk Calculation Tools

Of the two available risk calculation tools, four PCTs used QRISK2, one PCT used JBS2, and the remaining three used both depending on clinician choice and the clinical management system in use in GP practices (Table 5). Although both tools are recommended in the national guidance, there has been much debate about the relative strengths and weaknesses of these tools [7]. However, a recent independent validation found QRISK2 to be marginally less sensitive but more specific than JBS2; and to have stronger positive predictive power. The difference can result in QRISK2 reclassifying 43% of women and 45% of men previously deemed high risk (>20% in 10 years) by JBS2 into a low risk category (<20% in 10 years)[8]. This would be significant in reducing the cost and side-effects of unnecessary statin prescribing.

### Additional Interventions

There were differences across the eight PCTs in the way Health checks were used as a platform and opportunity to conduct other health interventions. For example, six PCTs used a questionnaire to detect alcohol-related problems; while four PCTs added a tool to measure physical activity (Table 5). Three of the eight PCTs also used the health check as an opportunity to do pulse examinations to screen for atrial fibrillation.

### Advice, Treatment and Referral

Once a person's level of CVD risk has been assessed, they should receive appropriate advice or treatment. All eight PCTs had explicit referral criteria and pathways, with most PCTs expecting GPs to actively manage high-risk patients. All PCTs also referred identified smokers to a smoking cessation service and referred or signposted appropriate patients to weight management and exercise programmes.

However, there was some variation in the way Health Checks were linked to non-medical support or services. One PCT commissioned a nurse coordinated multidisciplinary family based programme to provide integrated care for those at high risk of vascular disease within a community facility. Another used community-based health trainers to support high risk patients to undertake 'lifestyle changes'. Two other PCTs had commissioned programmes specifically for patients diagnosed with pre diabetes.

### Monitoring and Evaluation

There is some standardisation in the monitoring of the Health Checks Programme as all PCTs are currently required to submit a minimum dataset to NHS London (Table 6). Aside from this data, five PCTs reported collecting data on smoking status, alcohol consumption and levels of physical activity although there was variation in how this information was quantified. Three PCTs reported collecting data on 'advice given at the time of the health check' and four collected data on the 'number of referrals made'. Only four PCTS reported collecting data on screening outcomes such as the number of newly diagnosed cases of diabetes, hypertension, chronic kidney disease; and the number of patients initiated with statin therapy. Only one PCT was collecting data on the quality of checks.

**Table 6:** Current Health Check Data Set Required by DOH

**1**.	Number of people aged 40-74 eligible for an NHS Health Check
**2**.	Number of eligible people who have been offered an NHS Health Check.

**3**.	Number of eligible people who have received an NHS Health Check.

**4**.	% of eligible people who have been offered a NHS Health Check.

**5**.	% of eligible cohort who have received a NHS Health Check.

## Discussion

The findings from this survey demonstrate considerable variation in the implementation of a National Public Health Programme across eight PCTs within a single sector of London. The starkest finding was that when budgets were standardised per 100,000 eligible population there was an apparent twenty-fold difference between the least and best funded programme. This cannot be fully explained by a difference in health need across the 8 PCTs. However, when the outlying PCT with the largest spend was removed we see a strong negative correlation between the ranked index of deprivation and population-weighted budget (Table 3) indicating that PCTs responsible for the most deprived areas spent more per eligible person than PCTs responsible for more affluent areas. Interestingly a weak negative correlation was also found between CHD mortality rates in people under 75 years per 100,000 and population weighted budget. Such a degree of variation in funding is likely to result in differences in the impact of the programme at the population level.

The study also found other variations in the interpretation and implementation of national policy and guidance. A key consideration is whether these variations are appropriate and/or whether they contribute to a postcode lottery effect that is unfair or undesirable.

Universal screening of patients aged between 40 and 74 years is the strategy adopted by the DH to identify and pro-actively manage individuals at risk of CVD. Some experts have argued that targeting patients with known risk factors may be a more cost-effective [9] approach even though such an approach would identify fewer people at-risk [10]. In this study, all PCTs were mostly compliant with the national strategy although there was considerable variation in the approaches used to ensure population coverage.

Generally, a systematic as well as a targeted and opportunistic approach is required to ensure high and equitable population coverage for screening programmes [7]. A mix of locations can also be of value. For example, while General Practice is a logical setting on which to base systematic screening programmes, their limited accessibility outside common working hours can limit uptake. In addition some sections of the population (e.g. middle-aged, working class men) are resistant to taking up screening invitations from GP practices [11]. Offering alternative sites for screening in the community setting is therefore sensible, particularly involving pro-active methods to reach out to men and high-risk groups.

In this study, three PCTs based their Health Checks Programme entirely on GP practices, with no outreach or alternative locations although one of these had carried out health checks opportunistically at outreach events during the first year of the programme. The potential for such an approach to widen health inequalities is significant, given the fact that a proportion of working class men are both known to be resistant to preventative health measures provided at GP practices and at high risk of CVD [12].

The use of two different risk calculation tools may also result in differences in health outcome at the population level for reasons described above. In this instance, the variation is less the result of arbitrary or idiosyncratic differences as all PCTs were compliant with national guidance. However, the two recommended tools have different levels of specificity and sensitivity. Most importantly, QRISK2 will identify a smaller number of 'false positives' and will thereby reduce the incidence of iatrogenic harm caused by statins which increases the risk of liver dysfunction, acute renal failure, myopathy and cataract [13].

Another significant variation concerns the screening for diabetes. In theory, two thirds of type 2 diabetes can be prevented by lifestyle and diet interventions [14], and it is suggested that the Health Checks Programme could prevent 9,700 case of diabetes a year through the identification [a] of pre-diabetes and appropriate interventions [15].

There is however a lack of consensus about the best population-based approach for detecting undiagnosed diabetes and pre-diabetes. This is reflected in the national guidelines which do not make clear recommendations. RBG tests have been found to produce the lowest yield of new cases of diabetes. FBG tests are more sensitive and specific than RBGs (an FBG level of >5.5 mmol/l has a sensitivity and specificity of 80% and would lead to 20% of this sub-population requiring a definitive oral glucose tolerance test). However, FBG tests are more difficult to conduct and are also not effective at identifying people with pre-diabetes [13] (sensitivity 51.9%), therefore people with pre diabetes may be missed.

The FindRisc tool predicts a future diagnosis of diabetes using several factors that are simple to measure and non invasive. Using a cut off score of 11, FindRisc has a sensitivity of 66% in men and 77% in women in detecting diabetes [14]. QDSCORE uses another algorithm for estimating the risk of developing type 2 diabetes over a 10-year period although it is yet to be clinically validated [16].

The differences in the sensitivity and specificity of the various screening approaches and tests for diabetes will impact on the number of new cases of diabetes and pre diabetes identified through the Health Checks Programme. Given the lack of a standardised approach across the eight PCTs, this is likely in turn to result in differences in the effectiveness and efficiency of the programme.

The success of the Health Checks Programme is ultimately dependent not just on achieving high and targeted population coverage but also upon the quality of CVD risk management, including behaviour change interventions. Here it is evident that the quality, cost and effectiveness of such interventions vary considerably between PCTs (and are likely to vary within PCTs). For this reason, systems of evaluation that are capable of monitoring the quality of post-screening counselling, advice and clinical management are important.

Presently, there is strong guidance on the collection of a minimum national dataset. However, none of these data provide any information on quality or outcome and this survey found both varied and limited attempts by PCTs to collect data on the quality and impact of the Health Checks Programme, with only one PCT monitoring quality. Given that the monitoring and evaluation of quality and impact is an important ingredient of programmatic effectiveness in its own right, the lack and variability of monitoring and evaluation raises further concerns about the existence of a postcode lottery effect.

The survey also highlighted some variation which may not necessarily contribute to a postcode lottery effect. For example, variation in the payment made to GPs and pharmacies may not have a discernible impact on quality or outcomes, and may be explained in part by differences in the terms and conditions of contracts (e.g. whether or not consumables are supplied by the PCT). Similarly, we found differences in the availability of referral services and decisions taken to piggy-back additional public health interventions - but such differences cannot be assumed to result in differences in health outcomes without a broader understanding of other services and programmes provided by each PCT.

### Limitations

The main limitation of this study is that data on differences in the quality and impact of the Health Check Programme are not available. Another limitation of the study is that other programmes or initiatives to assess and manage CVD risks were not taken into consideration. For example, in one of the eight PCTs, a well-funded programme to manage CVD exists and has some overlap with the Health Checks Programme. Finally, a third limitation is that this study is focused on a small geographical area within London and is not representative of the country. However the fact that there are such large variations within a small area reinforces the likelihood of a considerable postcode lottery effect associated with the Health Checks Programme.

## Conclusion

The clinical policies and eligibility criteria for NHS screening programmes are usually uniform, standardised and evidence-based. Operational guidelines are provided to help avoid any postcode lottery effect, as well as to inform clinical providers with the latest evidence and to help ensure the best provision of care [17].

However, this study identifies a postcode lottery effect related to a national public health programme aimed at reducing the incidence of CVD. Furthermore, it shows this effect within a single geographical sub-sector of London, which may suggest even greater variation at a national level. The study raises questions about the balance between having nationally prescribed standards and guidelines (to prevent undesirable variation in practice or outcomes) and allowing local discretion and flexibility (to allow programmes to be tailored to local factors).

The study also suggests a lack of joint planning and sharing of information between PCTs within the same locality which could have helped minimise a postcode lottery effect, as well as achieved certain economies of scale (e.g. through the joint procurement of equipment). Certainly the findings provide sufficient grounds for the 8 PCTs to take steps towards greater harmonization, as well as to improve the quality of local monitoring and evaluation.

Although it is important to allow some flexibility in the design of the Health Checks Programme, some aspects of the programme might benefit from greater standardisation or stronger guidance. For example, it would seem beneficial to have clearer guidelines around the choice of risk calculator to estimate CVD risk and of screening approach for diabetes. Stronger guidance could also be supplied by the DH with regard to monitoring and evaluation that goes beyond the national minimum data set. Importantly, this would include guidance on the periodic (non-routine) collection of data on quality, as well as the use of participatory or action research methods that would promote continuous quality improvement.

This study therefore suggests a need for a more structured approach towards getting the balance right between nationally prescribed guidelines and standards that are mandatory, national guidance that is non-mandatory and programmatic elements that are left entirely to local discretion. The application of such an approach to the Health Checks Programme is shown in Table 7. While this approach may help minimise a postcode lottery effect, it should be noted that a trade-off between the benefits of complete standardisation and/or equity and the benefits of allowing local discretion and flexibility may be unavoidable.

**Table 7:** Programmatic Elements to be Nationally Prescribed, Encouraged Nationally, or Left to Local Discretion

	Nationally prescribed guidelines and standards that are mandatory	National guidance that is non-mandatory	Programmatic elements that are entirely left to local discretion
**Eligibility for Health Check**	Eligibility criteria in terms of age and ethnicity, with some allowance for clinical discretion		

**Approach to achieving coverage**	Stipulate use of both systematic and opportunistic approaches; as well as GP and non-GP settings (to avoid increasing inequity) Minimum coverage targets	Provide guidance on how systematic screening should be organised annually. Provide suggested targets for non-GP screening	Identification of non-GP settings and opportunistic approaches

**Funding and payment structure**		Guidance on payment rates per Health Check Suggested minimum and maximum cost of the programme per 100,000 population	

**Equipment and screening instruments**	CVD Risk Calculation Tool	POCT equipment that is accredited by MHRA Approach to diabetes and pre diabetes screening	Procurement of POCT.

**Additional Interventions**		Recommended additional screening tests that could be part of a Health Check Recommended method or tools for additional screening (e.g. AUDITc for alcohol screening)	Final selection of additional screening tests and tools

**Monitoring and evaluation**	Mandatory minimum dataset Minimum requirements for evaluation of quality and outcomes.	Guidance on suggested methods for evaluation of quality and outcomes. including an equity analysis	Final design of M&E strategy

**Referral Pathways**	Referral criteria and expectations for clinical conditions requiring medical attention	Best practice referral guidelines for weight management, smoking cessation, etc	Design of local referral pathways and commissioning of required services

## Competing interests

The authors declare that they have no competing interests.

## Authors' contributions

KM and CG designed the study methods and KM conducted all the primary data collection. DM conceived and supervised the study. KM, CG and DM all participated in the analysis of the data and the writing of the manuscript. All authors have read and approved the final manuscript.

## Endnotes

a. This is based on the assumption that checks would include all questions that make up the FINDRISC score and that people with a high FINDRISC score would receive an initial blood glucose test and those with a value over 6 would be called back to receive and oral glucose tolerance test.

b. This budget covers the costs of carrying out NHS Health Checks and relates to the costs of buying equipment to carry out the health checks, payments made to providers for carrying out checks and the costs of marketing the service.

c. Rank of deprivation out of 326 LA's nationally based on IMD scores in 2010 (most deprived to least)

d. Directly Standardised Rate of Coronary Heart Disease Mortality In Persons Aged under 75 per 100,000 population by PCT, 2006-2008 pooled [18]

e. MHRA Report 05051 2005: Polymer Technology Systems CardioChek PA Lipid System

f. MDA Evaluation Report 1995: Cholestech LDX Lipid Analyzer, MDA/95/23

## Pre-publication history

The pre-publication history for this paper can be accessed here:

http://www.biomedcentral.com/1471-2458/11/738/prepub

## Supplementary Material

Additional file 1 **Structured Questionnaire**. Structured questionnaire covered the following aspects of the Health Checks Programme: eligibility criteria; approach used to ensure population coverage; funding and associated payment structures; equipment and screening instruments; CVD risk calculation tool used; additional interventions; monitoring and evaluation; and advice treatment and referrals. Click here for file
