# Supplementary material for: Postcode Lotteries in Public Health - The NHS Health Checks Programme in North West London
Source: BMC Public Health. 2011 Sep 28;11:738. doi: 10.1186/1471-2458-11-738 (PMC3195760; doi:10.1186/1471-2458-11-738)
Supplement: Additional file 1 — Structured Questionnaire. Structured questionnaire covered the following aspects of the Health Checks Programme: eligibility criteria; approach used to ensure population coverage; funding and associated payment structures; equipment and screening instruments; CVD risk calculation tool used; additional interventions; monitoring and evaluation; and advice treatment and referrals. [file 1471-2458-11-738-S1.DOC]

**We are in the process completing a survey to look at the similarities and differences in Health Checks across the Sector please can you possibly complete the table below and return it to Kate May (**[**Katherine.May@hf-pct.nhs.uk**](mailto:Katherine.May@hf-pct.nhs.uk)**) by 16^th^ September. Thank you.**

| **Name of PCT** |  |
| --- | --- |
| **Opportunistic or Systematic Approach to NHS Health Checks Programme:** |  |
| **Health Checks are Carried out by:** |  |
| **Eligibility Criteria:** |  |
| **CVD Risk Calculator Used:** |  |
| **Diabetes Risk Screening Tool:** |  |
| **Data Collected:** |  |
| **Point Of Care Testing System Used:** |  |
| **GPPAQ Used:** |  |
| **Audit C:** |  |
| **Is AF screening carried out and if so how:** |  |
| **Is an IT solution being used to support Health Checks e.g. health diagnostics, Telehealth:** |  |
| **Individuals Identified as being high risk are referred to:** |  |
| **Total allocated budget for the programme 2010-2011:** |  |
| **Charge per Health Check:** |  |
| **Coverage Indicatators:** |  |
| **No of people invited for a Health Check to date** |  |
| **Number of Health Checks completed to date** |  |
| **Additional Information:** |  |
